# TRAF6 Is Required for Generation of the B-1a B Cell Compartment as well as T Cell-Dependent and -Independent Humoral Immune Responses

**DOI:** 10.1371/journal.pone.0004736

**Published:** 2009-03-09

**Authors:** Takashi Kobayashi, Tae Soo Kim, Anand Jacob, Matthew C. Walsh, Yuho Kadono, Ezequiel Fuentes-Pananá, Tomoko Yoshioka, Akihiko Yoshimura, Masahiro Yamamoto, Tsuneyasu Kaisho, Shizuo Akira, John G. Monroe, Yongwon Choi

**Affiliations:** 1 Molecular and Cellular Immunology, Medical Institute of Bioregulation, Kyushu University, Fukuoka, Japan; 2 Department of Pathology and Laboratory Medicine, University of Pennsylvania School of Medicine, Philadelphia, Pennsylvania, United States of America; 3 Department of Microbiology and Immunology, Keio University School of Medicine, Tokyo, Japan; 4 Department of Host Defense, World Premier International Immunology Frontier Research Center, and the Research Institute for Microbial Diseases, Osaka University, Osaka, Japan; University of Miami, United States of America

## Abstract

TNF receptor superfamily members, such as CD40 and the Toll-like receptors (TLRs), regulate many aspects of B cell differentiation and activation. TRAF6 is an intracellular signaling adaptor molecule for these receptors, but its role in B cells has not been clarified by previous genetic approaches, as the systemic deletion of the *TRAF6* gene results in perinatal lethality. Here we show that B cell-specific TRAF6 deficiency results in a reduced number of mature B cells in the bone marrow and spleen. Optimal T cell-dependent (TD) antigen responses, as characterized by isotype switching and long-lived plasma cell generation, are also impaired in B cell-specific TRAF6-deficient mice. B cell-specific TRAF6-deficient mice also exhibit lower levels of serum IgM and IgG2b and defective antigen-specific IgM production in response to T cell-independent (TI) antigens. Unexpectedly, TRAF6-deficient B cell progenitors are unable to generate CD5^+^ B-1 cells. These results reveal critical roles for TRAF6 in TD and TI humoral immune responses and in inductive fate decisions necessary to generate the B-1 B cell compartment.

## Introduction

Ligands for the Toll-like receptors (TLRs), such as lipopolysaccharide (LPS) and CpG-DNA, are powerful B cell mitogens, and they also induce proinflammatory cytokines such as interleukin (IL)-6 and the surface molecules CD40, B-7 and MHC class II [1], [2], [3], [4]. The tumor necrosis factor receptor (TNFR) superfamily member CD40 similarly induces not only clonal B cell expansion but also T cell-dependent (TD) responses, such as germinal center (GC) formation, antibody isotype switching, affinity maturation and differentiation into long-lived plasma cells [5], [6]. TNF receptor-associated factor 6 (TRAF6), a member of the TRAF family of cytoplasmic adaptors, transduces signals from the TNFRs [7] as well as from the TLRs [8], [9], thereby playing a critical role in innate immunity [10]. TRAF6 is recruited to the motif PXEXXAr/Ac, which is found in the IL-1 receptor-associated kinase (IRAK) adaptor molecules and in the cytoplasmic portion of TNFR family members like CD40 and receptor activator of NF-κB (RANK) [11], [12], [13]. TRAF6 mediates the activation of mitogen-activated protein (MAP) kinases such as p38, Erk and JNK, and NF-κB transcription factors.

TRAFs 1, 2, 3, 5 and 6 are recruited to specific domains in the cytoplasmic tail of CD40. The binding site for TRAF6 is distinct from that of other TRAFs (PXQXT motif), and there are structural differences between receptor recognition by TRAF6 and other TRAFs [13]. It has been shown that the various TRAFs have some unique and some overlapping functions *in vitro*; however, the roles of each TRAF downstream of CD40 in B cells are not fully understood. Recently, two separate groups examined transgenic mice with CD40 mutations that lack one or both TRAF binding sites [14], [15]. One study found that TRAF6 plays a critical role in antibody affinity maturation and the generation of plasma cells [14], whereas the other study concluded that TRAF6 has no discernible role in CD40-mediated activation and antibody class switching [15]. It is difficult to establish the importance of TRAF6 in B cell function from these two studies. The functional readouts examined were different; in addition, in one study the mutant CD40 molecule was widely expressed [14], while in the other it was expressed exclusively in B cells [15]. Finally, neither of these studies was designed to determine CD40-independent functions of TRAF6 in B cells.

There are difficulties associated with examining the role of different TRAFs in mature B cells using *TRAF* gene deletions. TRAF2-, 3-, 6-deficient mice die *in utero* or shortly after birth, suffering from multiple abnormalities in various organs. TRAF6-deficient mice develop osteopetrosis and occlusion of the bone marrow (BM) cavities from a lack of osteoclast function [16], [17]. The BM is an anatomically important site for early B cell development and for antibody production by plasma cells. Moreover, TRAF6-deficient mice lack lymph nodes [17] and therefore cannot support normal T cell-B cell interactions during the course of an immune response. Not unexpectedly, there are very few splenic B cells in these mice (approximately 5%, data not shown). Hence, analysis of the physiologic function of TRAF6 in B cells has not been amenable to genetic approaches.

In order to clarify the physiologic role of TRAF6 in the regulation of B cell development and function, we created B cell-specific TRAF6-deficient mice by crossing floxed TRAF6 mice (in which a *TRAF6* exon is flanked by *lox*P sites) with CD19-Cre mice. TRAF6 appears to influence several processes in B cell development and function. The mutant mice have fewer mature B cells in the BM and spleen and mount sub-optimal TD-antigen responses. Additionally, baseline IgM production as well as T cell-independent (TI) antigen-specific IgM production is severely impaired, most likely as a result of the complete absence of CD5^+^ B-1 cells (B-1a cells) in the peritoneal cavity. Our findings suggest novel functions for TRAF6, which appears to be required for both B-1 and B-2 cell homeostasis and to play a role in both in TI and TD humoral immune responses.

## Results

### Generation of B cell-specific TRAF6-deficient mice

**Figure 1:**
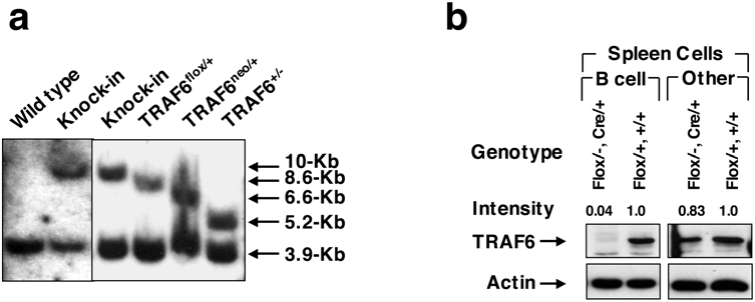
Generation of B cell specific TRAF6 KO mice. (a) Southern blot analysis to confirm the *Cre*-mediated deletion of the floxed fragment. (b) Western blot analysis to demonstrate specific deletion of TRAF6 in B cells. Lysates blotted with anti-TRAF6 or anti-actin from B cells and non-B cells are shown.

In order to study the physiologic functions of TRAF6 in B lymphocytes, we generated B cell-specific TRAF6-deficient mice (TRAF6-ΔB mice). To generate offspring in which *TRAF6* was deleted only in B cells, TRAF6^flox/flox^ mice [18] were crossed with CD19-Cre mice, in which the expression of Cre recombinase is driven by the CD19 promoter. B cell-specific deletion of the *TRAF6* gene was confirmed by polymerase chain reaction (data not shown) and western blot analysis (Fig. 1). Purified B cells from spleen of CD19^Cre/+^TRAF6^flox/flox^ mice contained almost undetectable levels of TRAF6 protein, although a substantial amount of TRAF6 was present in non-B splenocytes as well as in B cells from control mice. Similar results were obtained from CD19^Cre/+^TRAF6^flox/−^ mice (Fig. 1). Thus, CD19^Cre/+^TRAF6^flox/flox^ and CD19^Cre/+^TRAF6^flox/−^ mice were used interchangeably. TRAF6-ΔB mice were born at the expected Mendelian ratio and exhibited normal growth rates, without inflammatory lesions or osteopetrosis.

### TRAF6 deficiency in B cells results in defective proliferation, IL-6 production and signaling in response to TLR ligands and anti-CD40

**Figure 2:**
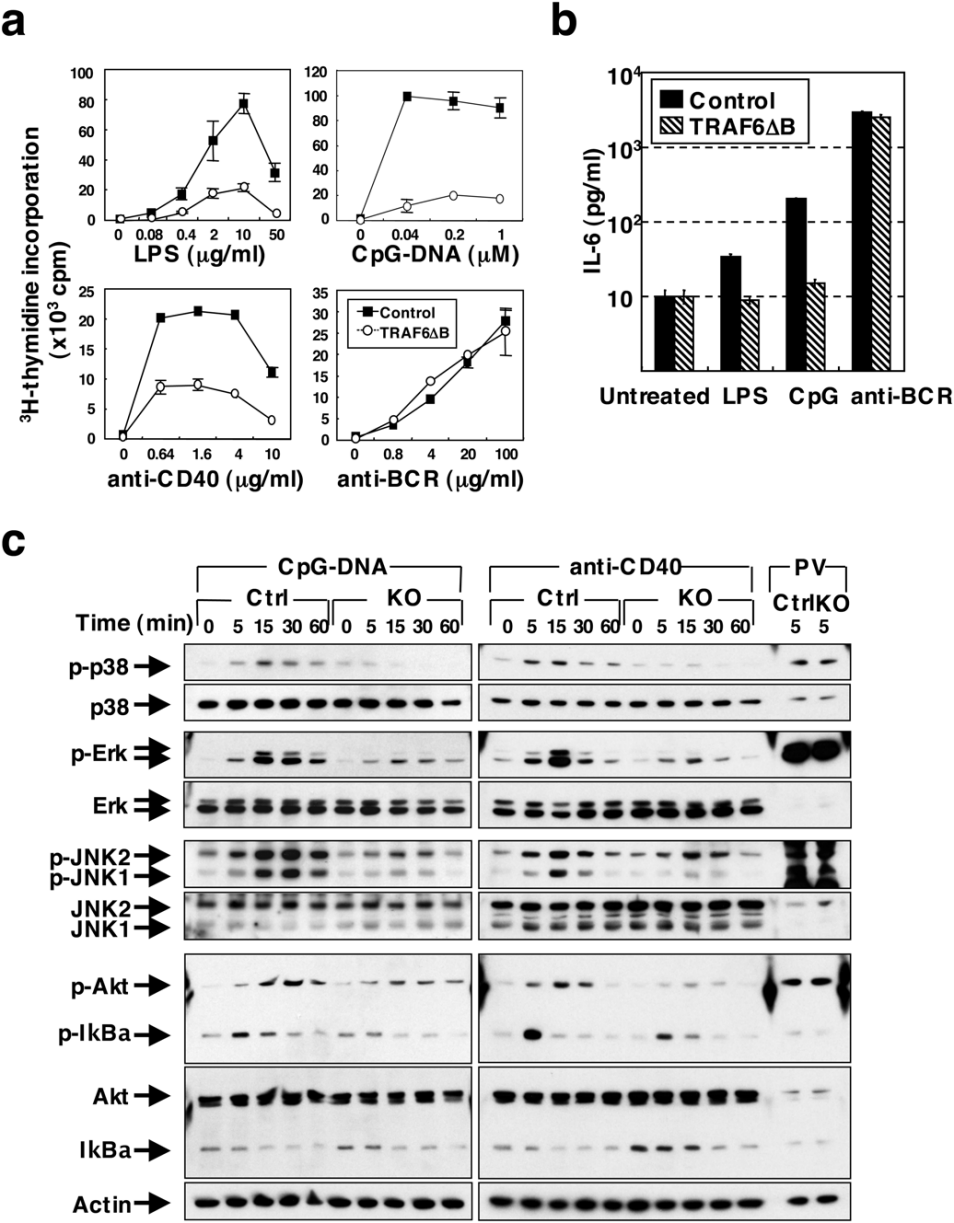
Defective proliferation, IL-6 production and signal activation of TRAF6-deficient B cells in response to TLR ligands and anti-CD40 antibody. (a) Proliferative response of splenic B cells from control or TRAF6-ΔB stimulated *in vitro* with varying doses of LPS, CpG-DNA, anti-CD40 antibody or anti-BCR antibody. Results are representative of at least 4 different experiments. Data are presented as mean±SD. (b) IL-6 production in media supernatants from splenic B cells cultured for 2 days with LPS (10 µg/ml), CpG-DNA (1 µM) or anti-BCR antibody (20 µg/ml). (c) Western blot analysis of phosphorylation of the indicated proteins in lysates of splenic B cells stimulated *in vitro* with CpG-DNA (4 µM), anti-CD40 antibody (8 µg/ml) or pervanadate (PV) for the indicated times. Sequential immunoblots with antibodies to the phosphorylated (indicated by “p-”) and total protein are compared. Results are representative of at least 4 different experiments.

TLRs and CD40, which stimulate B cells upon interaction with their respective ligands, induce the recruitment of TRAF6 [1], [4], [5], [6]. Therefore, we first examined proliferation of splenic B cells from TRAF6-ΔB mice *ex vivo* in response to LPS, CpG-DNA, anti-CD40 Ab and anti-BCR Ab (Fig. 2a). Proliferation of TRAF6-ΔB B cells in response to LPS, CpG-DNA and anti-CD40 Ab was severely impaired, while proliferation induced by BCR crosslinking was comparable to the control B cells. In addition, LPS- and CpG-DNA-induced production of IL-6 was nearly abolished in the TRAF6-ΔB B cells (Fig. 2b).

Stimulation by TLR ligands or CD40 ligation causes the phosphorylation and activation of MAP kinases as well as phosphorylation and degradation of IκBα [1], [6]. Control B cells stimulated with CpG-DNA induced phosphorylation of p38, Erk, JNK, Akt and IκBα, as well as IκBα degradation within 15 min. However, these events were significantly impaired in B cells from TRAF6-ΔB mice (Fig. 2c, left panels). Notably, phosphorylation of p38 was undetectable in TRAF6-deficient B cells. Similar results were observed in B cells stimulated with anti-CD40 Ab (Fig. 2c, right panels) or LPS (data not shown). These results suggest that TRAF6 mediates signals from TLRs and CD40 that regulate B cell proliferation and cytokine production.

### TRAF6 is critical for mature B cell fate determination

**Figure 3:**
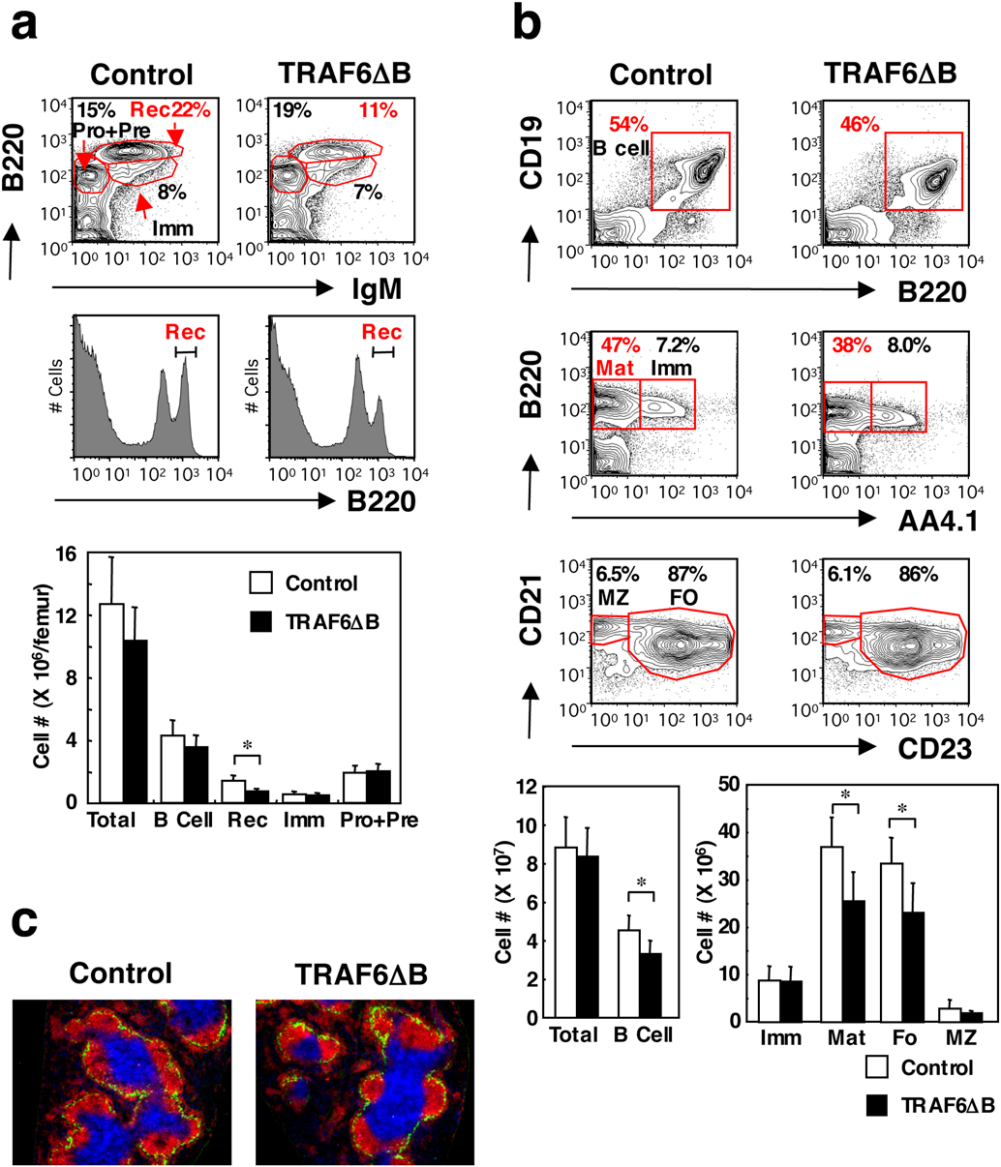
TRAF6 is required for mature B cell homeostasis. (a) Flow cytometric analysis of BM from control and TRAF6-ΔB mice stained with the indicated antibodies (top-most and middle panels). The percentages of encircled areas are indicated. Pro+Pre, pro-B and pre-B cells; Imm, immature B cells; Rec, recirculating B cells. Results are representative of 5 mice. Lowest panel, absolute cell numbers; *, *p*<0.005. (b) Similar analysis of total spleen cells. Total B cells are shown in the topmost panels; mature (Mat) and immature (Imm) B cells are compared in the middle panels. The lowest panels compare the follicular (FO) and MZ sub-populations within the mature B cell gate shown in the middle panels. Cell numbers of each B cell subset in the spleen are shown in the lowest panels. Results are representative of at least 4 mice; *, *p*<0.05. (c) Splenic microarchitecture visualized by immunohistochemical staining with anti-B220 (red), anti-CD3 (blue) and anti-MOMA1 (green) antibodies. Results are representative of at least 5 mice.

We next examined B cell development and homeostasis in TRAF6-ΔB mice. The early developmental subsets of B cells in the BM of TRAF6-ΔB mice were comparable to control mice (Fig. 3a, top panels), with no obvious difference in pre- and pro-B cell subsets (data not shown). The ratios of B220^low^IgM^+^IgD^−^ immature B cells were also comparable between TRAF6-ΔB and control mice (Fig 3a, top panel).

In marked contrast to early B cell development in the BM, B220^high^IgM^+^IgD^+^ mature recirculating B cells were significantly diminished in TRAF6-ΔB mice (12.8±1.3% in TRAF6-ΔB compared to 20.2±2.3% in controls, Fig. 3a, top and middle panels). This deficiency is reflected in the absolute cell numbers (Fig. 3a, bottom). In the spleen, the percentage of B cells (CD19^+^B220^+^) was also significantly reduced in TRAF6-ΔB mice (39.2±7.1% compared to 50.3±4.8% in the control mice, Fig. 3b, upper panels; absolute numbers shown in the lowest panel). The reduction was more prominent in the IgM^low^IgD^high^AA4.1^−^ mature B cells (Fig. 3b, middle panels) than in the IgM^high^IgD^low^AA4.1^+^ transitional B cells, which are their developmental precursors [19]. This finding suggests that TRAF6 regulates the late step of mature B cell development or homeostasis. Within the mature subset, the ratios of the CD21^+^CD23^+^ follicular B cell subset and CD21^high^CD23^−^ marginal zone (MZ) B cell subset were almost identical between TRAF6-ΔB and control mice. Furthermore, immunohistochemical (IHC) analysis of spleen sections from TRAF6-ΔB mice revealed normal splenic architecture, with typical T cell areas surrounded by follicular and MZ B cell areas, separated by MOMA1^+^ metallophilic macrophages (Fig. 3c).

### Cell survival and alternative NF-κB signaling are intact in TRAF6-deficient B cells

**Figure 4:**
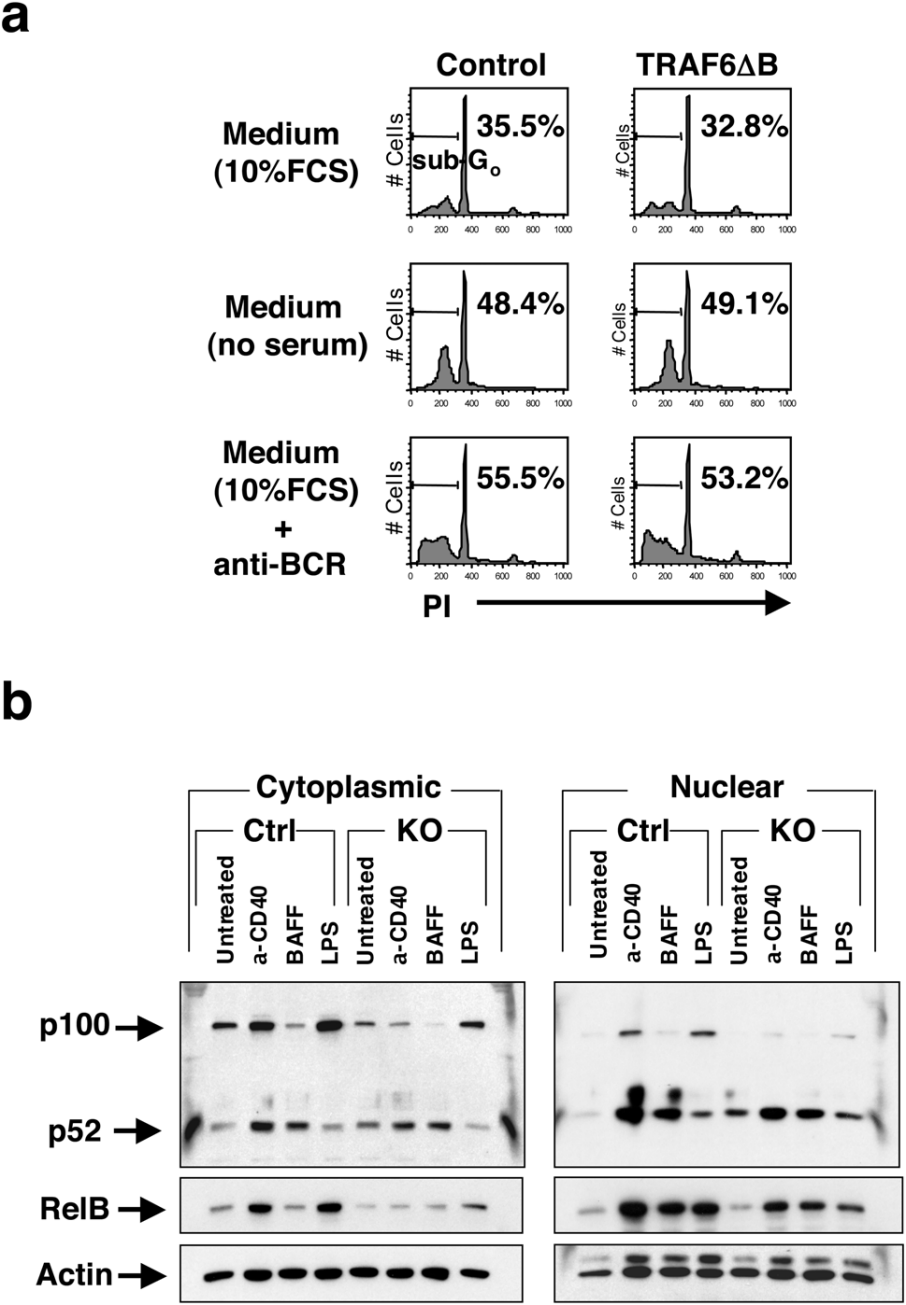
Alternative NF-κB signal is intact in TRAF6-deficient B cells. (a) Survival of splenic B cells from control or TRAF6-ΔB mice cultured *ex vivo* in various conditions indicated. Percentages of apoptotic B cells (sub-G0 peaks) are indicated. (b) Cytoplasmic fractions and nuclear fractions from splenic B cells stimulated *in vitro* with anti-CD40 antibody (4 µg/ml), recombinant BAFF (2 µg/ml) or LPS (10 µg/ml) for 24 hr immunoblotted with anti-p100/52, anti-RelB and anti-actin antibodies. Results are representative of 2 independent experiments.

Since mature B cell numbers in TRAF6-ΔB mice were reduced, we examined *ex vivo* survival of splenic B cells from TRAF6-ΔB and control mice cultured with or without serum or in the presence of anti-BCR Ab. Both control and TRAF6-deficient B cells showed similar percentages (approximately 30%) of apoptotic B cells (sub-G0 peak) after 14 hr in the presence of serum (Fig. 4a). This percentage was increased to approximately 50% by serum depletion or BCR crosslinking. Similar results were obtained when transitional B cells isolated from sub-lethally irradiated TRAF6-ΔB and control mice were used (data not shown). These results suggest that TRAF6 deficiency does not affect B cell survival or susceptibility to apoptosis, arguing that the defects observed were a consequence of impaired fate determination rather than homeostasis.

**Figure 5:**
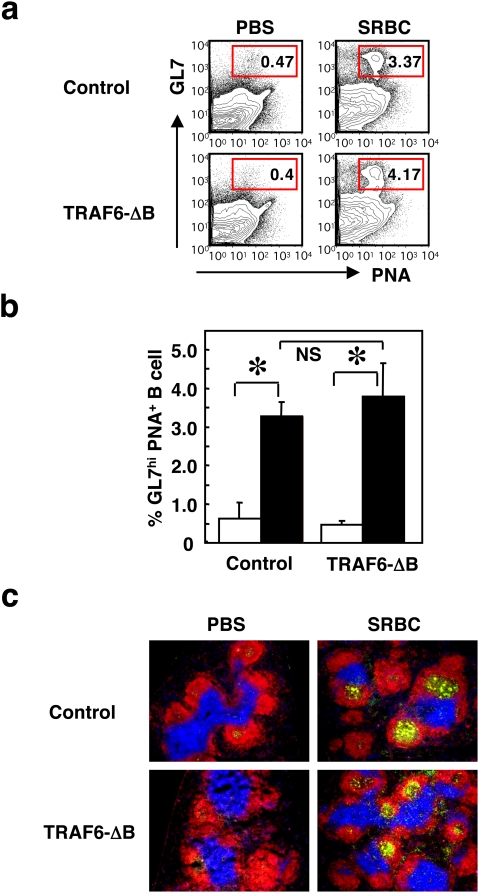
Germinal center formation in response to TD antigen is intact in TRAF6-ΔB mice. (a) GC formation in the spleen in response to PBS or sheep erythrocyte (SRBC) immunization examined by flow cytometric analysis using fluorescent PNA and antibodies to B220, CD19 and GL7. Profiles of PNA vs. GL7 in B220^+^CD19^+^ B cell populations are shown. Ratios of GL7^hi^ and PNA^+^ B cells in the encircled areas are indicated. (b) Frequencies of GL7^hi^ and PNA^+^ B cells are quantified. Data are presented as mean±SD of five samples of one representative experiment out of 2 independent experiments. (c) Spleen sections were examined for GC formation by immunohistochemical staining with fluorescent PNA (green) and antibodies to B220 (red) and CD3 (blue).

The TNF family member B cell activating factor (BAFF, also known as BLyS) and its cognate receptor BAFF-R are required for generation and maintenance of the mature B cell pool [20], [21], [22]. It has been reported that signals from BAFF-R activate the alternative NF-κB pathway via processing of the NF-κB2 protein p100 to p52 [23], [24]. Correspondingly, western blot analysis of control B cell lysates demonstrated that BAFF induced processing of p100 to p52 but did not upregulate p100 or RelB in control B cells, suggesting specific activation of the alternative NF-κB pathway (Fig. 4b). Consistent with previous reports [23], [24], CD40 ligation induced processing of NF-κB2 as well as upregulation of p100 and RelB, indicating the activation of both canonical and alternative pathways, whereas LPS treatment induced only the canonical pathway. As shown in Fig. 5b, B cells from TRAF6-ΔB mice exhibited normal processing of p100 to p52 in response to BAFF and anti-CD40 Ab. However, upregulation of p100 and RelB was obviously impaired in response to both LPS and anti-CD40 Ab, suggesting defective canonical NF-κB activation. This finding is consistent with the observed defect in IκBα phosphorylation and degradation (Fig. 2). Therefore, TRAF6 is selectively required for canonical NF-κB activation upon TLR or CD40 stimulation but not for activation of the alternative pathway downstream of CD40 or BAFF-R. Again, these results point towards a role for TRAF6 in B cell subset specification rather than in generating survival signals for homeostasis.

### Impaired TD antigen-specific IgG production and long-lived plasma cell generation in TRAF6-ΔB mice

TD humoral immune responses, including TD antigen-specific IgG production, GC formation, isotype switching, affinity maturation and generation of memory B and antibody-producing plasma cells, do not develop in mice lacking CD40 or CD154 [25], [26]. Given that CD40 signaling is impaired in the TRAF6-ΔB mice (Fig. 2), we examined TD immune responses. TRAF6-ΔB and control mice were immunized intraperitoneally with sheep erythrocytes and examined by flow cytometry and IHC analysis for the formation of GCs. Flow cytometric analysis of spleen cells from TRAF6-ΔB and control mice stained for the GC markers peanut agglutinin (PNA) and GL7 [27] revealed that GL7^+^PNA^+^ GC B cells were induced at comparable ratios (3.80±0.86% in TRAF6-ΔB vs. 3.28±0.27% in control mice, Fig. 5a and b). Moreover, IHC analysis showed that PNA^+^ GCs were present in the B cell areas in both TRAF6-ΔB and control mice (Fig. 5c), indicating that TRAF6 is not required for GC formation induced by TD antigens.

**Figure 6:**
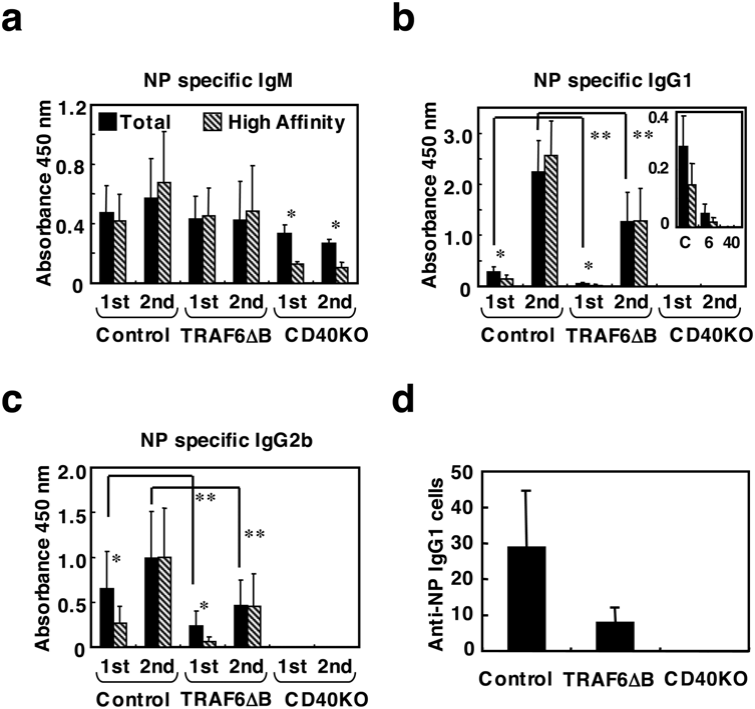
TD antigen-specific immunoglobulin and long-lived plasma cell production are impaired in TRAF6-ΔB mice. (a–c) Defective antigen-specific IgG1 and IgG2b production in response to TD antigens for primary response to the TD Ag NP-KLH (“1^st^”, 7days after immunization) and also for the secondary response (“2^nd^”, 14 days after immunization as detailed in Methods). The level of high-affinity (clear bars) and total (black bars) Abs are compared: (a), antigen-specific IgM levels; (b), IgG1; (c), IgG2b. Inset in (b) shows the primary response plotted on a smaller scale. Data are presented as mean±SD. *; *p*<0.05, compare total and high affinity Igs, **; *p*<0.05, compare control and TRAF6-ΔB mice. (c) Reduced number of long-lived plasma cells producing NP-specific IgG1 in the BM of TRAF6-ΔB mice collected 60 days after immunization (as described in a–c), detected by ELISpot assay.

TRAF6-ΔB, CD40-deficient and control mice were also immunized with a separate TD antigen, NP-KLH, and the primary responses were measured 7 days later. NP-specific IgM production was comparable to control and CD40-deficient mice (Fig. 6a). Levels of NP-specific IgG1 and IgG2b were diminished as compared to those in control mice, though still higher than in the CD40-deficient mice (Fig. 6b and c). During the secondary response measured 14 days after boosting, the levels of NP-specific IgG1 and IgG2b were augmented in the TRAF6-ΔB mice, though still lower than in the control (Fig. 6b and c). These results suggest that antigen-specific isotype switching still occurs in the absence of TRAF6, albeit inefficiently.

We also examined affinity maturation by comparing the level of antibodies captured by NP_30_-BSA against those captured by NP_3_-BSA in an enzyme-linked immunosorbent assay (ELISA), as described previously [14]. About half or less of the total antigen-specific IgG1 and IgG2b was high-affinity Ig during the primary response in the control mice; in the secondary response, almost all the IgG1 and IgG2b antibody was high affinity (Fig. 6b and c). Although total antigen-specific IgG1 and IgG2b levels were lower in TRAF6-ΔB mice, the proportion of high-affinity antibody was identical to that in the control during secondary responses, suggesting that TRAF6 is not required for affinity maturation, possibly because other TRAFs downstream of CD40 can compensate for the lack of TRAF6.

We also measured the terminal differentiation of B cells into plasma cells. The numbers of long-lived antibody-secreting cells in the BM of TRAF6-ΔB, CD40-deficient and control mice were compared by enzyme-linked immunospot (ELISpot) assay (Fig. 6d). The number of plasma cells secreting NP-specific IgG1 was reduced in TRAF6-ΔB mice, indicating that TRAF6 plays a role in this terminal differentiation step. Our results indicate that TRAF6 is selectively required for specific CD40 functions during the response to TD antigen.

### Reduced serum IgM and IgG2b levels and defective TI antigen-specific IgM production in TRAF6-ΔB mice

**Figure 7:**
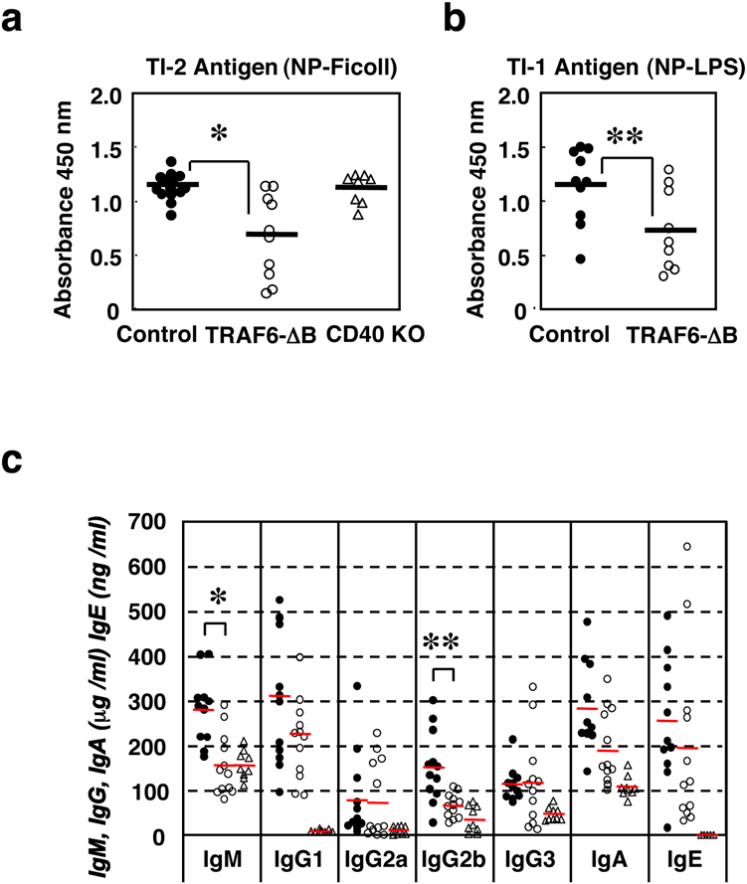
Reduced serum IgM and IgG2b levels and defective TI antigen-specific IgM production in TRAF6-ΔB mice. (a, b) Defective antigen-specific IgM production in response to the TI antigens NP-Ficoll (TI type 2, a) or with NP-LPS (TI type 1, b). *: *p*<0.004; **: *p*<0.04. (c) Serum immunoglobulin levels from unimmunized 14-week-old control (closed circle), TRAF6-ΔB (open circle) and CD40 KO (open triangle); control; n = 11, TRAF6-ΔB; n = 12, CD40 KO; n = 5; *: *p*<0.001 and **: *p*<0.005.

We next examined whether TRAF6 also regulates TI immune responses. TRAF6-ΔB, CD40-deficient and control mice were challenged with either the TI type I antigen NP-LPS or the type II antigen NP-Ficoll. One week after immunization, NP-specific IgM production in TRAF6-ΔB mice was significantly lower than in control or CD40-deficient mice in response to both antigens (Fig. 7a and b), indicating that TI immune responses in TRAF6-ΔB mice are poor.

Consistent with defects in TI immune responses, TRAF6-ΔB mice also showed a reduced level of circulating antibody at steady states. When the basal levels of the various classes of Ig in the sera of unimmunized mice were measured by isotype-specific ELISA, TRAF6-ΔB mice exhibited significantly lower levels of IgM and IgG2b compared to control mice (Fig. 7c).

### TRAF6 is required for B-1a cell development in the peritoneal cavity

**Figure 8:**
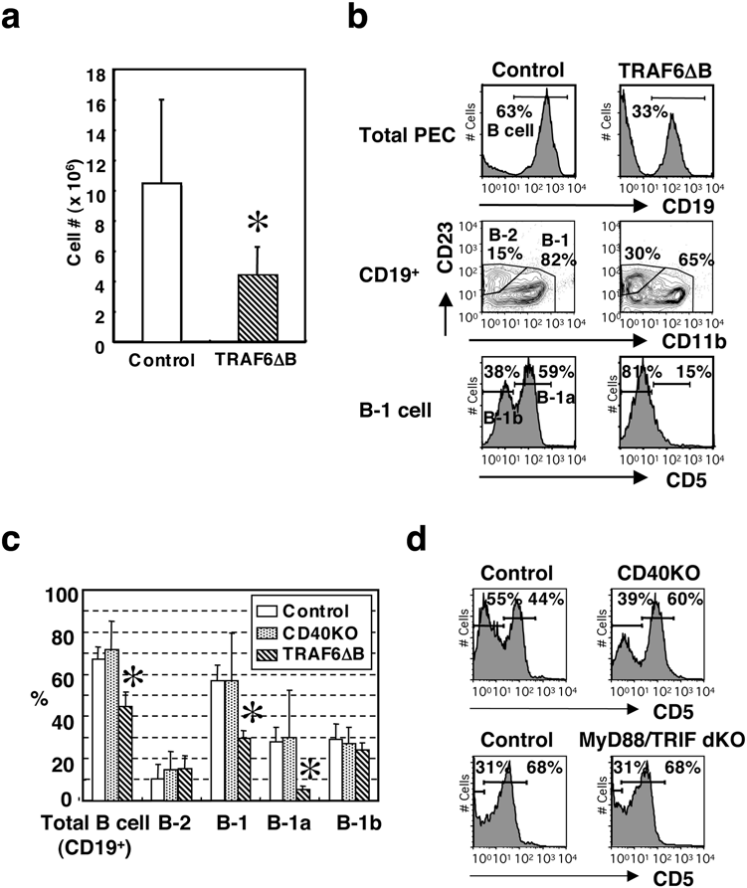
Peritoneal CD5^+^ B cell population is absent in TRAF6-ΔB mice. (a) Total peritoneal exudate cells (PECs) numbers from control and TRAF6-ΔB mice. (control; n = 5, TRAF6-ΔB; n = 5) *: *p*<0.05 (b) Peritoneal exudate cells (PECs) from control and TRAF6-ΔB mice analyzed by flow cytometry. Total CD19^+^ B cells (top panels) are subdivided into B-1 and B-2 subsets (middle panels), and the CD23^−^CD11b^+^ B-1 subset is further divided into B-1a and B-1b (bottom panels). Results are representative of at least 8 mice. (c) The ratios of each B cell subset in the PECs from control, CD40 KO and TRAF6-ΔB mice are shown. Data are presented as mean±SD. (control; n = 12, CD40 KO; n = 4, TRAF6-ΔB; n = 8) *: *p*<0.01 compare to control. (d) Profiles of CD5 expression in B-1 cells from CD40-deficient mice and MyD88/TRIF-doubly deficient mice are analyzed as described in (b).

B-1a cells are thought to potentiate responses to certain TI antigens and/or to be the major source of circulating antibody (which is primarily IgM) in the absence of overt antigen exposure [28], [29]. Therefore, we first examined whether B-1a cell development is affected in TRAF6-ΔB mice. The overall number of cells in the peritoneal exudates of the TRAF6-ΔB mice was about 40% less than the control (Fig. 8a). The ratio of total CD19^+^ B cells was significantly reduced in the peritoneal exudate cells of TRAF6-ΔB mice (44.9±6.6% of total cells compared 67.2±5.9% in the control, Fig. 8b, upper panels). Within B cell populations, the proportion of the CD11b^+^CD23^−^ B-1 cell subset in TRAF6-ΔB mice was particularly affected, with the proportion of CD11b^−^CD23^+^ B-2 cells being correspondingly higher (Fig. 8b, middle panels). Since the total B cell number was reduced in TRAF6-ΔB mice, the percentage of B-2 cells in the total peritoneal exudate was identical to that of control mice (Fig. 8c). Most notably, the CD5^+^ B-1a [29] cells within the B-1 compartment were almost completely absent (Fig. 8b, lower panels). Because both CD19^Cre/+^TRAF6^+/+^ and CD19^+/+^TRAF6^flox/flox^ control mice showed identical ratios of CD5^+^ B-1a cells in the peritoneal exudate (data not shown), the deficiency in TRAF6-ΔB mice could be attributed to *TRAF6* deficiency rather than to haploinsufficiency of the *CD19* gene. We hypothesized that if either CD40 or the TLRs regulate B-1a cell development through TRAF6, we would see a similar phenotype in the corresponding knockout mouse. However, neither CD40-deficient nor MyD88/TRIF-doubly deficient mice had any deficiency in B-1a cells (Fig. 8d). This intriguing finding suggests that unique TRAF6-dependent receptors are crucial for B-1a cell regulation.

## Discussion

Here we have investigated the importance of the signaling adaptor TRAF6 to B cell development and function. Our previous study emphasized the role of TRAF6 in innate immunity [10]; however, the role of TRAF6 in lymphocytes and adaptive immunity has not been established. Because of the breadth and severity of the *TRAF6*-deficient phenotype [16], [17], we generated B cell-specific TRAF6-deficient (TRAF6-ΔB) mice.

Our results demonstrate a broad role for TRAF6 in B lymphocyte development and function. Mature B cell populations in the BM and spleen are reduced, though the pro-, pre- and immature B cells in the BM and transitional B cells in the spleen are of normal numbers (Fig. 3). Although the proliferative response of TRAF6-deficient B cells induced by TLR ligands and CD40 ligation was severely impaired, BCR crosslinking induced normal proliferation (Fig. 2a). Moreover, this impairment does not appear to be the result of increased susceptibility to apoptosis (Fig. 4). It has been shown recently that BAFF-induced survival plays an important role in the maintenance and homeostasis of the peripheral B cell pool [20], [21], [22]. However, we show that signaling from BAFF-R was normal in TRAF6-ΔB cells (Fig. 4), suggesting that yet-to-be-elucidated molecules activate TRAF6-mediated signals required for generation or maintenance of the mature B cell pool. These results thus suggest a previously unexpected complexity in the nature of signals necessary for these processes.

CD40 signaling is known to be important in certain antigen-driven phenomena in TD immune responses [25], [26]. In conjunction with the defect in CD40 signaling *in vitro* (Fig. 2), we found isotype switching to IgG1 and IgG2b in response to TD antigen was severely impaired, as was the generation of long-lived plasma cells (Fig. 6). Intriguingly, other CD40-dependent processes, such as affinity maturation and GC formation, were unaffected. This finding indicates that a specific subset of CD40 functions operate through TRAF6.

Our most surprising observation is the complete lack of CD5^+^ B-1a cells in the peritoneal cavity of TRAF6-ΔB mice (Fig. 8), which is accompanied by lowered basal levels of serum IgM or “natural” antibody and sub-optimal TI immune responses (Fig. 7). Both phenomena are postulated to be largely dependent on peritoneal B-1 cells and MZ B cells [29]. Mutations of positive regulators of BCR signaling, such as PKCβ, PKCγ, PI-3K, BLNK, Vav-1 and CD19, are known to result in a reduction in B-1 cells [29]. Since there were no defects in BCR responses *in vitro* (Fig. 2), we were concerned that the B-1a defect may be the result of CD19 haploinsufficiency of the TRAF6-ΔB mice, which are CD19^Cre/+^TRAF6^flox/flox^ or CD19^Cre/+^TRAF6^flox/−^. However this was not the case, since CD19^Cre/+^TRAF6^+/+^ mice exhibit normal ratios of peritoneal B-1a cells. It has been shown that the combined loss of the NF-κB subunits p50 and c-Rel diminishes the peritoneal CD5^+^ B-1a cell population [30]. Given that the canonical NF-κB pathway is impaired in TRAF6-ΔB mice (Figs 2 and 4), it appears that a TRAF6/NF-κB pathway is essential for the development of peritoneal CD5^+^ B-1a cells. Curiously, neither CD40-deficient nor MyD88/TRIF-doubly deficient (thus defective in TLR signaling) mice have impaired B1-a cell development (Fig. 8c). These results suggest that signals from a receptor(s) other than CD40 or TLRs regulate B-1a cell development or that CD40 and TLRs compensate for each other's loss. The importance of BCR function in B-1 cell development has long been appreciated [29]. However, the involvement of TLRs and other receptors for bacterial products is controversial. It was demonstrated that the administration of LPS or the presence of enteric bacteria is necessary for the development of B-1 cells in anti-RBC autoantibody-transgenic mice [31]. Future experiments will be necessary to identify which cell surface receptors are necessary for TRAF6-mediated B-1 cell development.

We have demonstrated that TRAF6 is involved in a wide variety of B cell processes. It is crucial for certain CD40 effector functions in B cells during TD humoral immune responses and also for the generation of optimal TI immune responses. In addition, TRAF6 is necessary for B-1a cell development and for the maintenance of the mature B cell pool. Understanding the regulation of this adaptor protein therefore will contribute to our knowledge of B cell function in the development of the immune system and during its response to antigen.

## Materials and Methods

### Mice

Generation of floxed TRAF6 knock-in ES cells has been described [10]. The *neo* cassette flanked by *lox*P sites in the knock-in ES cells was deleted by pMC-Cre transfection *in vitro*; this deletion was confirmed as described [10] and by Southern blot analysis. Mutant ES clones were injected into C57BL/6 blastocysts. CD19-Cre mice have been described [32] and were obtained from Dr. Alexander Tarakhovsky at the Rockefeller University; CD40 KO mice were purchased from The Jackson Laboratory. MyD88/TRIF double KO mice have been described previously [33]. All animals are maintained in accordance with the applicable portions of the Animal Welfare Act and the DHHS, Guide for the Care and Use of Laboratory Animals.

### *In vitro* proliferation and cytokine production

Splenic B cells purified by using anti-B220 MACS MicroBeads (Miltenyi Biotec) resulted in a preparation of >95% CD19^+^ cells. For B cell proliferation assays, 1×10^5^ purified B cells/well were cultured with or without LPS (Sigma), CpG-DNA [34], anti-CD40 antibody (HM40-3, BD Pharmingen) or F(ab′)_2_ anti- mouse Ig Ab (Jackson Immuno Research Laboratories) for 2 days. 0.5 µCi of ^3^H-thymidine was pulsed for the last 10 hr, and incorporation was measured by liquid scintillation counting. For cytokine production assays, IL-6 in the supernatant of similar cultures stimulated with LPS (10 µg/ml), CpG-DNA (1 µM) or F(ab′)_2_ anti-Ig antibody (20 µg/ml) was determined using an OptEIA mouse IL-6 ELISA set (BD Pharmingen).

### Immunoblots

Purified splenic B cells pre-incubated in Hank's balanced salt solution (HBSS) at 37°C for 30 min were stimulated with CpG-DNA (4 µM), anti-CD40 Ab (8 µg/ml) or pervanadate for various times and then lysed with lysis buffer containing 1% Triton X-100 and protease and phosphatase inhibitors. For alternative NF-κB signaling, cells were stimulated with anti-CD40 Ab (4 µg/ml), recombinant BAFF (2 µg/ml, Pepro Tech) or LPS (10 µg/ml) for 24 hr. Cytoplasmic and nuclear fractions were extracted using a Nuclear Extraction kit (Active Motif). Protein samples were separated by 10% sodium dodecyl sulfate polyacrylamide gel electrophoresis (SDS-PAGE) and transferred onto Immobilon-P PVDF membrane, blocked with 5% or 2.5% milk in PBS with 0.1% Tween-20, incubated with primary Abs overnight at 4°C followed by horseradish peroxidase (HRP)-conjugated secondary Abs for 1 hr. Sources of antibodies were as follows: p38, Erk, JNK, Akt, IkBα, p-p38, p-Erk, p-Akt, p-IkBα (Cell Signaling Technology); p-JNK (BD Transduction laboratories); RelB (Santa Cruz); TRAF6 (MBL); and actin (Sigma). Rabbit anti-mouse p100/52 polyclonal antibody was generously provided by Dr. Amer A. Beg (Columbia University, New York). Blots were developed with ECL substrate (Amersham) and exposed to Kodak BioMax XAR film.

### Flow cytometry

Antibodies against CD3 (145-2C11), CD5 (53-7.3), CD11b (M1/70), CD16/32 (2.4G2), CD19 (1D3), CD21 (7G6), CD23 (B3B4), CD40 (HM40-3), B220 (RA3-6B2), IgM (R6-60.2) and GL7 were purchased from BD Pharmingen; mAb AA4.1 was purified from the hybridoma [35] kindly provided by Dr. David Allman at the University of Pennsylvania and conjugated to APC by standard techniques. Single-cell suspensions were incubated with anti-CD16/32 (Fc-block) and stained with FITC-, PE-, APC- and biotin-conjugated antibodies, followed by incubation with streptavidin-PerCP-Cy5.5 (Pharmingen). Flow cytometric analysis was performed on a FACSCalibur flow cytometer (BD biosciences) using CellQuest (BD biosciences) and FlowJo (Tree Star, Inc.) software.

### ELISA for serum Ig measurement

For Fig. 7c, sera were collected from 14-week-old control, TRAF6-ΔB and CD40 KO mice. Goat anti-mouse Ig (H+L) Ab was used for capture, and isotype-specific HRP-conjugated anti-mouse Ig Abs were used for detection. These and Ig standards were purchased from Southern Biotech Associates Inc.

For Figs 6 and 7a and 7b, NP-specific IgM, IgG1 and IgG2b in the sera were measured by capture with NP_30_-BSA (total antigen-specific Igs), or NP_3_-BSA (high-affinity Igs) followed by detection with isotype-specific HRP-conjugated anti-mouse Ig Abs.

### Immunizations

NP-BSA, NP-KLH, NP-Ficoll and NP-LPS were purchased from Biosearch Technologies Inc. 12-16-week-old control, TRAF6-ΔB and CD40 KO mice were immunized intraperitoneally with NP_67_-Ficoll (10 µg/mouse) for TI type-2 immune responses or with NP_1_-LPS (5 µg/mouse) for TI type-1, and serum Igs were measured after 1 week. For primary TD immune responses, mice were immunized intraperitoneally with alum-precipitated NP_30_-KLH (200 µg/mouse), and sera were collected 7 days later. For the secondary response, these mice were boosted intraperitoneally with antigen (50 µg/mouse) 14 days after immunization, and sera were collected 14 days after this boost. For GC formation, mice were immunized intraperitoneally with sheep erythrocytes without adjuvant.

### ELISpot assay

Long-lived antibody-producing plasma cells were detected by ELISpot assay using a Protein Detector ELISpot Kit (Kirkegaard & Perry Laboratories, Inc.). Mice were immunized with NP-KLH as described above. 60 days after immunization, BM cells were harvested and plated in triplicate at 2×10^6^ cells/well in NP-BSA coated 96-well PVDF plates, which were incubated for 4–6 hr at 37°C in a 5% CO_2_ incubator and then washed with PBS containing 0.05% Tween-20. Spots were developed with HRP-conjugated anti-mouse IgG1 followed by the TrueBlue chromogen substrate, and they were counted under a dissection microscope or with an ELISpot plate reader.

### GC formation

Ten days after immunization with sheep erythrocytes, spleens were collected and subjected to flow cytometry and IHC. For flow cytometry, single-cell suspensions were stained for B220, CD19, GL7 and PNA (Vector Laboratories). For IHC, frozen sections were stained with anti-CD3-PE-Cy5, anti-B220-PE-TexasRed and PNA-FITC or anti-MOMA1-FITC (Serotec).

### Statistical analysis

For statistical analysis, we used Student's *t*-test. A 95% confidence limit was considered significant and is defined as *p*<0.05.
